# What data collection methods work best for COVID19 outbreak surveillance for people with end stage kidney disease? An observational cohort study using the UK Renal Registry

**DOI:** 10.1186/s12882-023-03148-8

**Published:** 2023-05-08

**Authors:** Shalini Santhakumaran, Manuela Savino, Fran Benoy-Deeney, Retha Steenkamp, James Medcalf, Dorothea Nitsch

**Affiliations:** 1 UK Renal Registry, Brandon House Building 20A1 Southmead Road Filton, Bristol, BS34 7RR UK; 2 Great Western Hospital NHS Foundation Trust, Swindon, UK; 3 University of Leicester, Leicester, UK; 4 Leicester General Hospital, Leicester, UK; 5 London School of Hygiene and Tropical Medicine, London, UK; 6 Royal Free London NHS Foundation Trust, London, UK

**Keywords:** COVID-19, ESKD, Surveillance, Infection, Dialysis, KRT

## Abstract

**Background:**

Patients on kidney replacement therapy (KRT) are vulnerable to severe illness from COVID-19. Timely, accurate surveillance is essential for planning and implementing infection control at local, regional and national levels. Our aim was to compare two methods of data collection for COVID-19 infections amongst KRT patients in England.

**Methods:**

Adults receiving KRT in England were linked to two sources of data on positive COVID-19 tests recorded March-August 2020: (1) submissions from renal centres to the UK Renal Registry (UKRR) and (2) Public Health England (PHE) laboratory data. Patient characteristics, cumulative incidence by modality (in-centre haemodialysis (ICHD), home HD, peritoneal dialysis (PD) and transplant), and 28-day survival were compared between the two sources.

**Results:**

2,783/54,795 patients (5.1%) had a positive test in the combined UKRR-PHE dataset. Of these 2,783, 87% had positive tests in both datasets. Capture was consistently high for PHE (> 95% across modalities) but varied for UKRR (ranging from ICHD 95% to transplant 78%, *p* < 0.0001). Patients captured only by PHE were more likely to be on transplant or home therapies (OR 3.5 95% CI [2.3–5.2] vs. ICHD) and to be infected in later months (OR 3.3 95%CI [2.4–4.6] for May-June, OR 6.5 95%CI [3.8–11.3] for July-August, vs. March-April), compared to patients in both datasets. Stratified by modality, patient characteristics and 28-day survival were similar between datasets.

**Conclusions:**

For patients undergoing ICHD treatment the collection of data submitted directly by renal centres allows constant monitoring in real time. For other KRT modalities, using a national swab test dataset through frequent linkage may be the most effective method. Optimising central surveillance can improve patient care by informing interventions and assisting planning at local, regional and national levels.

**Supplementary Information:**

The online version contains supplementary material available at 10.1186/s12882-023-03148-8.

## Introduction

The COVID-19 pandemic has been, and still is, a challenge for the public health surveillance systems monitoring its evolution at national, regional, and local levels. Such surveillance systems provide vital information on the populations most at risk, highlight any observed inequalities and inform the interventions needed to mitigate the spread of the disease in the population [1].

People requiring kidney replacement therapy (KRT) are often immunocompromised or have multiple comorbidities and are therefore particularly vulnerable following COVID-19 infection [2, 3]. In addition, given that in-centre haemodialysis (ICHD) requires the regular treatment (usually three times a week) of multiple patients in the same area, and the ensuing repeat exposure to infection, HD centres were considered a high-risk area from the outset of the pandemic [4]. This necessitated increased efforts to monitor infections and introduce measures to reduce the risk of transmission.

In the early stages of the pandemic, there was a need to quickly plan and implement infection control measures to protect these vulnerable patients whilst maintaining access to their life-saving kidney treatments. This highlighted the requirement for timely surveillance of infections in dialysis centres, especially for planning both transport and dialysis stations for affected patients. Prior to the introduction of COVID-19 vaccinations, the key infection control measures against nosocomial outbreaks included a combination of active surveillance for early case detection, isolation of suspected cases, and contact tracing to identify potential secondary cases. These measures are less effective if testing is restricted to symptomatic individuals, as the pathogen SARS-CoV-2 responsible for COVID-19 may cause asymptomatic infection, and such infections may contribute to a significant proportion of transmission. Therefore, screening of all hospital admissions and outpatient visits prior to a scheduled admission had become necessary. In some centres, this had led to the introduction of wide screening and monitoring of patients treated with ICHD [5], starting in May 2020 for most London renal centres[6] though screening policies varied by hospital trust and region [7]. Consequently, renal centres may have been aware of any test results for patients on ICHD, including asymptomatic cases. This may not have been the case for other modalities where no such wide screen-testing protocols were in place, rather shielding was advised to minimise exposure [8].

Monitoring of the pandemic among patients on KRT can be done by a range of agencies, from the public health surveillance units who get notified of positive test results, to renal centres collating their own data to plan care. The UK Renal Registry (UKRR) is an established national registry collecting a range of data on all patients receiving KRT from all renal centres in the UK. Starting at the end of March 2020, the UKRR additionally received weekly data on patients with COVID-19 on KRT from renal centres in the UK to facilitate local planning [9]. A combination of staff sickness absence and dialysis demand from COVID-19 patients on intensive care wards led to increasingly stretched facilities. The UKRR commenced monitoring to enable weekly management of resources in terms of staff time, patient transport and dialysis equipment between renal centres at regional level. The data allowed infection control recommendations from the UK Kidney Association [10] and the Kidney Quality Improvement Partnership [7] to be regularly updated to reflect the changing course of the pandemic. In addition, Public Health England (PHE) received all positive test results in their role of monitoring and addressing the outbreak in England. These data were subsequently shared with the UKRR during the first wave of the pandemic but were only available for occasional linkage.

The UKRR’s work in collecting data from multiple sources allows for a comparative analysis to help understand challenges in real-time data collection when monitoring the COVID-19 outbreak and outcomes of patients on KRT treatment. The aim of this study is to compare the patient characteristics, cumulative incidence by modality and 28-day survival between the two sources to inform monitoring of future outbreaks.

## Materials and methods

### Study cohort

All patients receiving KRT in England on 31 December 2019 were extracted from provisional quarterly returns submitted to the UKRR by renal centres. These contain a wide range of clinical and demographic variables that are provided from the centre’s IT system. Data are validated and cleaned by the UKRR to produce a finalised database on an annual basis. Patients with no National Health Service (NHS) numbers were omitted, as were those on the national opt-out register, as this prevented linkage to the PHE database. Patients who died before 1 March 2020 were not included as they died before the period covered by the UKRR COVID-19 dataset. The Demographics Batch Service (a tracing facility provided by NHS Digital) was used to link the cohort to the latest NHS death data using NHS number and date of birth. These patients were then linked using NHS numbers to both the UKRR and PHE COVID-19 datasets to give the study cohort of patients with a positive test recorded in either source.

### COVID-19 data submitted by renal centres (UKRR COVID-19 database)

The NHS number, date of birth and date of test for patients who tested positive for COVID-19 were returned weekly from renal centres in the UK for polymerase chain reaction (PCR) tests occurring from 3 March 2020. In this study we included tests from 1 March 2020–31 August 2020 to match the PHE data. Centres were asked to return data on all COVID-19 test positive patients, regardless of symptoms, however in the early phase of the pandemic tests were only done in symptomatic patients. Renal centres established their own data capture, usually using a spreadsheet of known patients who were known to have COVID-19 when treated at the centre, which staff added to if they were made aware of other cases (e.g. a patient being admitted to another hospital and a copy of the discharge letter being passed on to the responsible nephrologist). In later phases of the epidemic outbreak (around May 2020) increased availability of tests allowed centres to screen patients irrespective of symptoms.

### COVID-19 data from PHE (PHE linked database)

Patient identifiers from the study cohort were linked to the COVID PCR test data by PHE as part of a wider match of UKRR patients. Data were linked twice during the study period, with the latest tests up to 31 August 2020. Details of the methodology used by PHE are described on the UK Department of Health and Social Care website [11]. Any patients opting out of participation in research were not included in the data returned by PHE. Returned data items used in this study were the earliest positive specimen date and pillar (pillar 1: swab testing in PHE labs and NHS hospitals for those with a clinical need, and healthcare workers; pillar 2: swab testing for the wider population as per government guidance).

### Ethics

The UKRR holds data on kidney patients under Sect. 251 of the NHS Act (2006), granted by the Health Research Authority’s Confidentiality Advisory Group. This gives the UKRR permission to carry out analyses on de-identified data without individual patient consent. Patients can opt out from data linkage using a national opt-out system. Collection of COVID-19 data from renal centres and data linkage with PHE was done under the Control of Patient Information (COPI) notice for COVID-19 research.

### Data analysis

The number and percentage of COVID-19 patients captured by each source (UKRR COVID-19 and PHE linked) was described by clinical and demographic characteristics from UKRR data (KRT treatment modality, age, sex, area-level deprivation (Index of Multiple Deprivation (IMD) rank quintile calculated from patient postcode [12]), ethnicity, whether they were on the waiting list for a transplant on 31 December 2019), and COVID-19 test data (the month of the positive test and the testing pillar (clinical settings (pillar 1) or community settings (pillar 2)). The cumulative number of positive COVID-19 tests (first positive test in each patient only) was shown by source overall and separately for each modality. For tests captured in both sources the difference in test date (the date the swab was taken) was described. For differences in proportions, *p-*values are from the chi-squared test. Survival to 28 days by modality was illustrated using Kaplan-Meier plots. Multinomial logistic regression was used to examine which clinical and demographic characteristics were associated with which data source the positive test was captured by (PHE only, UKRR only, or both sources, with the latter as the reference group). In contrast with the previous analyses this allowed us to compare between data source groups as there was no overlap. Analysis was performed in SAS v9.4 [13].

## Results

**Fig. 1:**
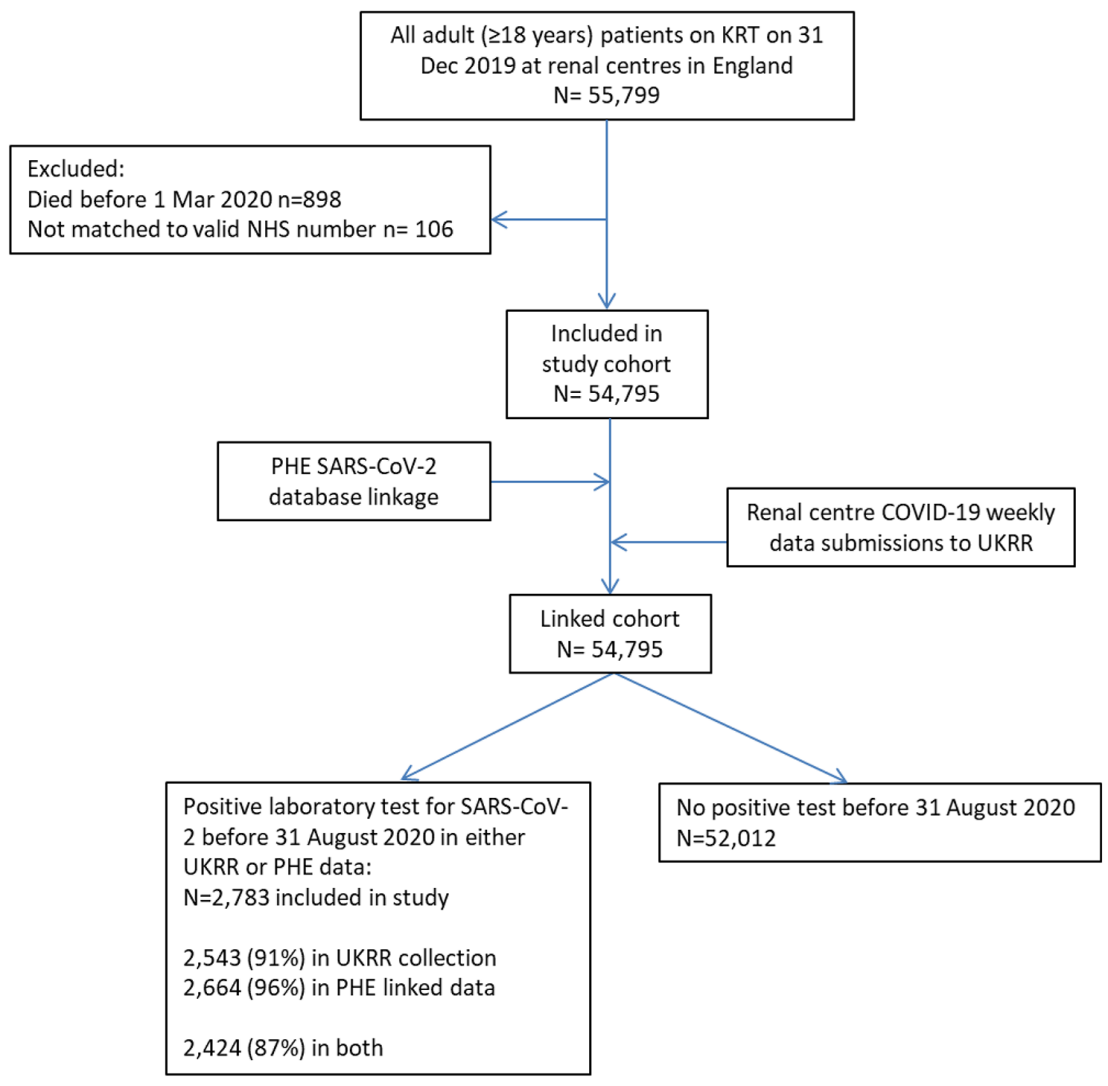
Flowchart for study cohort

There were 54,795 adult patients on KRT in England, as at 31 December 2019 who were still alive on 1 March 2020 and were available for linkage to the PHE database (Fig. 1). Of these patients 2,783 (5.1%) had a positive laboratory test for SARS-CoV-2 in the combined UKRR-PHE data source. The proportion of people on ICHD was much higher in patients testing positive for COVID-19 (76%, n = 2,114) than it was in the whole study cohort (36%, n = 19,541). In comparison, the percentage on each modality in the whole study cohort was 57% for transplant, 5.5% for PD and 2.1% for home HD.

**Fig. 2:**
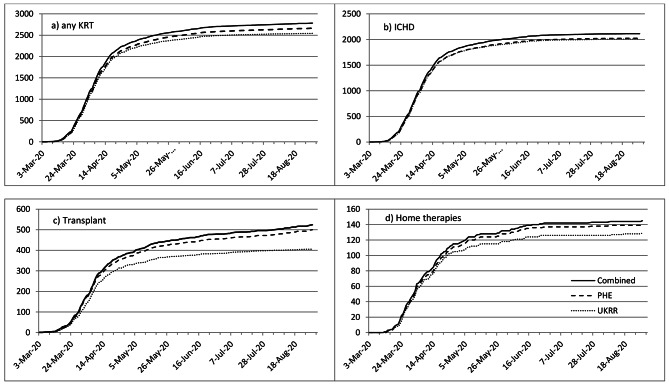
Cumulative number of positive COVID-19 tests by source (UKRR collection submitted by renal centres, linkage with Public Health England (PHE) or the combination of these) and test date, for patients on (a) any kidney replacement therapy (KRT), b) in-centre haemodialysis (ICHD), c) transplant and d) home therapies (HT, home haemodialysis or peritoneal dialysis)

The overall agreement was good, with 2,424 (87%) cases appearing in both sources. 2,543 cases (91%) appeared in the UKRR data and 2,664 (96%) in the PHE data. Data capture in the PHE dataset was consistent across modalities (Table 1), with over 95% of cases captured. In the UKRR dataset, 95% of patients on ICHD were captured, falling to 78% for transplant patients, 89% of PD patients and 88% of home HD patients (*p* < 0.0001). During the first six weeks of the pandemic the two sources captured a similar number of cases overall, but beyond that the numbers diverged, for transplant and home therapies patients (Fig. 2). The UKRR database captured a decreasing proportion of cases as the first wave progressed, from 95% in March to 39% in August (Table 1, p < 0.0001).

**Table 1:** Capture of positive COVID-19 tests by source of positive test data (UKRR, PHE or combined) and patient characteristics

	Combined	UKRR	PHE	In both sources
	N	n (%)	n (%)	n (%)
Total	2783	2543 (91.4)	2664 (95.7)	2424 (87.1)
Modality				
In-centre haemodialysis	2114	2008 (95)	2024 (95.7)	1918 (90.7)
Transplant	524	406 (77.5)	500 (95.4)	382 (72.9)
Peritoneal dialysis	104	93 (89.4)	101 (97.1)	90 (86.5)
Home haemodialysis	41	36 (87.8)	39 (95.1)	34 (82.9)
Age group (years)				
18–39	183	155 (84.7)	170 (92.9)	142 (77.6)
40–59	806	729 (90.5)	764 (94.8)	687 (85.2)
60–79	1384	1277 (92.3)	1337 (96.6)	1230 (88.9)
80+	410	382 (93.2)	393 (95.9)	365 (89)
Sex				
Male	1734	1589 (91.6)	1663 (95.9)	1518 (87.5)
Female	1049	954 (90.9)	1001 (95.4)	906 (86.4)
Area-level deprivation				
1 = least deprived quintile	281	255 (90.8)	270 (96.1)	244 (86.8)
2	380	344 (90.5)	369 (97.1)	333 (87.6)
3	567	526 (92.8)	544 (95.9)	503 (88.7)
4	744	682 (91.7)	714 (96)	652 (87.6)
5 = most deprived quintile	811	736 (90.8)	767 (94.6)	692 (85.3)
Ethnicity				
White	1417	1280 (90.3)	1358 (95.8)	1221 (86.2)
Asian	663	607 (91.6)	631 (95.2)	575 (86.7)
Black	494	460 (93.1)	476 (96.4)	442 (89.5)
Mixed	59	53 (89.8)	55 (93.2)	49 (83.1)
Other	80	75 (93.8)	77 (96.3)	72 (90)
Missing	70	68 (97.1)	67 (95.7)	65 (92.9)
Waitlisted (dialysis only)				
Not listed	1902	1799 (94.6)	1825 (96)	1722 (90.5)
Listed	357	338 (94.7)	339 (95)	320 (89.6)
Month of positive test				
March	902	855 (94.8)	875 (97)	828 (91.8)
April	1419	1326 (93.5)	1360 (95.8)	1267 (89.3)
May	266	215 (80.8)	250 (94)	199 (74.8)
June	121	102 (84.3)	107 (88.4)	88 (72.7)
July	39	31 (79.5)	38 (97.4)	30 (76.9)
August	36	14 (38.9)	34 (94.4)	12 (33.3)
Pillar^1^				
Pillar 1	2609	2407 (92.3)	2609 (100)	2407 (92.3)
Pillar 2	55	18 (32.7)	55 (100)	18 (32.7)
Missing	119	119 (100)	0 (0)	

^1^ Pillar was only provided in the PHE data

**Fig. 3:**
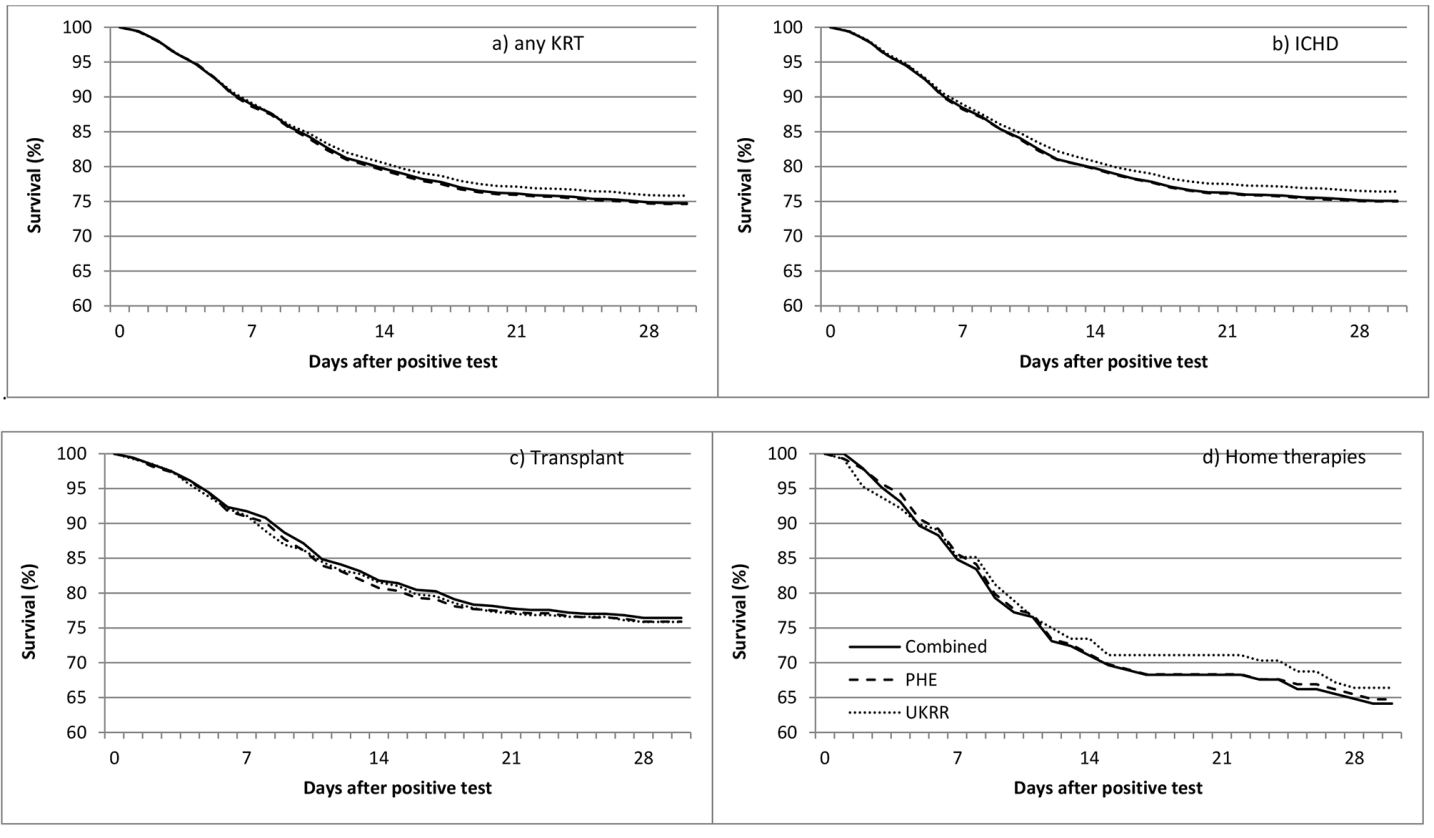
Kaplan-Meier plot of survival to 28 days after positive test, by source (UKRR collection submitted by renal centres, linkage with Public Health England (PHE) or the combination of these), for patients on (a) any kidney replacement therapy (KRT), b) in-centre haemodialysis (ICHD), c) transplant and d) home therapies (HT, home haemodialysis or peritoneal dialysis). For the analysis using all sources, if the test dates in UKRR and PHE differed, the earliest was used

The percentage of cases captured by both sources increased with age, from 78% in 18–39 year olds to 89% in those aged 80 and over (Table 1, p < 0.0001). Although only 55 infections from Pillar 2 community testing were reported, only a third of these were also found in the UKRR database, compared to 92% of Pillar 1 infections. There were little differences in capture by sex, area-level deprivation, ethnicity, or by whether the patient was on the transplant waiting list at the end of 2019 (dialysis patients only). After stratifying by treatment modality, the distribution of the patient characteristics was very similar for both data sources (supplementary Table 1). Patterns of 28-day survival were similar for both data sources, including for transplant patients where data capture in the UKRR dataset was lower (Fig. 3).

Results from the multinomial model (Table 2) show which factors were associated with being in the PHE dataset only, or the UKRR dataset only, compared to being in both datasets (adjusting for all factors simultaneously). Patients who were captured in only the PHE dataset were more likely to be on transplant or home therapies (OR 3.5 95%CI [2.3–5.2] vs. ICHD) and be infected in later months (OR 3.3 95%CI [2.4–4.6] for May-June, OR 6.5 95%CI [3.8–11.3] vs. March-April), compared to patients appearing in both datasets. Patients who were captured in only the UKRR dataset were more likely to be young (OR 2.2 95%CI [1.1–4.2] for 18–39 years vs. 60–79 years) and infected in May-June compared to March-April (OR 2.3 95%CI [1.5–3.7]) but these were not part of patterns over age and test dates.

**Table 2:** Results from multinomial model for source of COVID-19 data being PHE only or UKRR only compared to being in both, adjusting for modality, sex, index of multiple deprivation, ethnicity, age and test date period

	PHE only (n = 240) vs. both (n = 2,424)	UKRR only (n = 119) vs. both (n = 2,424)
Modality	Odds ratio (95% CI)	
ICHD	1	1
Tx + HT^1^	3.46 (2.33,5.15)	1 (0.57,1.76)
Sex		
Male	1	1
Female	1.08 (0.81,1.44)	1.13 (0.77,1.66)
IMD quintile		
1 - least deprived	1	1
2	0.94 (0.53,1.64)	0.63 (0.26,1.51)
3	0.75 (0.43,1.31)	0.92 (0.43,1.94)
4	1.05 (0.62,1.78)	0.96 (0.46,2.00)
5 - most deprived	1.09 (0.65,1.83)	1.27 (0.63,2.56)
Ethnicity		
White	1	1
Asian	0.73 (0.51,1.06)	1.08 (0.68,1.71)
Black	0.85 (0.55,1.30)	0.79 (0.45,1.40)
Mixed/Other^1^	0.83 (0.42,1.65)	1.11 (0.49,2.53)
Age group		
18–39	1.3 (0.79,2.14)	2.17 (1.11,4.23)
40–59	0.96 (0.68,1.35)	1.51 (0.96,2.37)
60–79	1	1
80+	1.36 (0.85,2.18)	1.37 (0.77,2.46)
Period		
March-April	1	1
May-June	3.32 (2.38,4.62)	2.34 (1.49,3.67)
July-August	6.53 (3.79,11.26)	1.41 (0.42,4.75)
Waitlisted or transplanted		
No	1	1
Yes	1.34 (0.89,2.03)	1.13 (0.67,1.92)

1 Groups combined due to small numbers captured by UKRR only. CI – Confidence interval, Tx – Transplant, HT -Home therapies (peritoneal dialysis and home haemodialysis)

For patients that were captured in both the UKRR and PHE sources, the agreement in the test date was high, with only 193 (8%) of cases having more than two days difference in test dates. There were more discrepant cases later in the period, with 7.5% of cases having more than 2 days difference in date in March-April, increasing to 9.8% in May-June and 19% in July August (*p* = 0.01). Date discrepancies of more than 2 days were found in around 7% of COVID-19 cases for ICHD and home therapies and 10.7% for transplant (*p* = 0.09).

## Discussion

Overall agreement between the COVID data submitted to the UKRR by renal centres, and that recorded by PHE was good, with 87% of the COVID-19 cases amongst the 2019 prevalent KRT population in England appearing in both data sources. The PHE dataset was a more reliable source for monitoring the number of infections amongst transplant and home therapies patients, and those occurring later during the second wave of the pandemic.

A key strength of this study is the use of the UKRR database which allowed the definition of a cohort to link to COVID-19 data, and contains demographic and clinical information, allowing comparison of the patient characteristics of the COVID-19 cases in the two data sources. Further linkage via NHS Digital provided mortality data beyond the latest available in the UKRR database. This study is limited to a retrospective comparison of the two data collections for a fixed cohort. A comprehensive evaluation of the wider surveillance processes would be valuable but was beyond the scope of the study. Data on testing frequency were not available. It is likely that there was higher testing frequency in the ICHD population [14], so cases were more likely to be detected than for other modalities.

Tests performed outside a hospital setting (e.g. community health services), while few in number during the study period, were less likely to be reported by renal centres compared to those done in clinical settings. Despite these differences, once stratified by modality, the characteristics of the patients were similar, as were their survival patterns. This suggests that transplant and home therapies patients captured in the UKRR dataset may be representative of the wider population, and inferences about the survival of these patients are generalizable. However further exploration using modality-specific analysis of those not captured in the UKRR dataset was not possible due to small numbers. For patients tested outside the renal centre, the test date reported by the UKRR may not be the exact date the swab was taken. Nonetheless, the test dates recorded in both sets were very similar, and the incidence was very similar in both sources over the first six weeks of monitoring. Monitoring the progression of the pandemic could be affected by the lower rates of agreement in the later months, though overall infection rates were lower at this time.

PHE data are available for England only and limited by the linkage frequency. The data from PHE are provided by linking identifiers for existing patients and are therefore limited to patients already known to the UKRR, which at the time of linkage did not include patients commencing KRT in 2020. Linking data to external sources requires data-sharing agreements to be in place and can be time-consuming, resulting in delayed reporting and a lag in the cohort available for linkage. In contrast, for most of the pandemic all UK renal centres submitted data weekly, to be published within days of submission. The UKRR dataset gives a more complete picture of the impact of COVID-19 on ICHD patients as it includes the latest patients with up-to-date data on treatment modality.

A strength of the UKRR COVID-19 data collection is the provision of national weekly surveillance in the KRT population. A similar approach was taken in France, where the national REIN registry of patients on KRT was adapted to include COVID-19 data collection, facilitating national weekly updates and reporting by region [15]. Other large-scale projects include the European Renal Association COVID-19 Database (ERACODA) [16] and the United States Renal Data System (USRDS) COVID-19 report [17], but neither are geared towards active surveillance through rapid reporting. Local surveillance reports have helped support local mitigation strategies, but these cannot necessarily be extrapolated nationally due to regional variation [18]. Contemporary reports are essential for surveillance and beneficial for management of renal services. Nonetheless, regular submission of COVID-19 data is a considerable burden for the renal centres, along with the additional data processing that occurs at the UKRR. The PHE data are obtained through a more automated process, though this can have drawbacks. Examination of cases reported to the UKRR by renal centres but not submitted to PHE found that a large number were occurring at one centre, which had a technical flaw that prevented test results being submitted to PHE. By submitting the data themselves, renal centres confirm the number of cases which is not currently part of the process with the PHE data.

## Conclusion

This analysis shows that surveillance of infections with population coverage of a specific group of patients can be difficult to achieve with only one data collection methodology. The possibility of integrating different data sources is the most efficient way to obtain data that are timely but also accurate. In our experience, for the KRT population, linking PHE data, data submitted by renal centres and the UKRR database of patients undergoing KRT treatment has allowed us to obtain more detailed information than is often available through surveillance alone, and to compare two different surveillance systems to outline their advantages and disadvantages in the context of the specific renal replacement treatment modality. This comparative analysis shows that while for patients undergoing ICHD treatment the collection of data submitted directly by renal centres is accurate and allows constant monitoring in real time, for other KRT modalities the possibility to use a national swab tests dataset through frequent linkage may be the most effective method, thus reducing the workload of the renal centres in collecting these data. Further linkage with hospital data would allow further exploration of other outcomes such as hospitalisation, ICU admission and ventilator use.

## Electronic supplementary material

Below is the link to the electronic supplementary material.

Supplementary Material 1

## Data Availability

The data underlying this article are available from the UKRR through the UKRR’s data application process – see https://ukkidney.org/audit-research/how-access-data. For any data access queries, contact ukrr-research@renalregistry.nhs.uk.

## Abbreviations

HD: Haemodialysis
ICHD: In-centre haemodialysis
KRT: Kidney replacement therapy
NHS: National Health Service
PD: Peritoneal dialysis
PHE: Public Health England
UKRR: UK Renal Registry
