# Supplementary material for: What data collection methods work best for COVID19 outbreak surveillance for people with end stage kidney disease? An observational cohort study using the UK Renal Registry
Source: BMC Nephrol. 2023 May 8;24:130. doi: 10.1186/s12882-023-03148-8 (PMC10166021; doi:10.1186/s12882-023-03148-8)
Supplement: Supplementary file 1 — Supplementary Material 1 [file 12882_2023_3148_MOESM1_ESM.pdf]

Supplementary table 1: Characteristics of people on kidney replacement therapy with COVID-19, by modality and source of COVID-19 test data.

	Home therapies				ICHD				Transplant			
	UKRR		PHE		UKRR		PHE		UKRR		PHE	
	N	%	N	%	N	%	N	%	N	%	N	%
Total	129	100.0	140	100.0	2008	100.0	2024	100.0	406	100.0	500	100.0
Sex												
Male	85	65.9	95	67.9	1249	62.2	1258	62.2	255	62.8	310	62.0
Female	44	34.1	45	32.1	759	37.8	766	37.9	151	37.2	190	38.0
Area-level deprivation												
1 = least deprived quintile	18	14.0	18	12.9	190	9.5	195	9.6	47	11.6	57	11.4
2	20	15.5	23	16.4	257	12.8	259	12.8	67	16.5	87	17.4
3	40	31.0	40	28.6	404	20.1	404	20.0	82	20.2	100	20.0
4	23	17.8	29	20.7	556	27.7	561	27.7	103	25.4	124	24.8
5 = most deprived quintile	28	21.7	30	21.4	601	29.9	605	29.9	107	26.4	132	26.4
Ethnicity												
White	76	58.9	84	60.0	1002	49.9	1028	50.8	202	49.8	246	49.2
Asian	20	15.5	20	14.3	479	23.9	469	23.2	108	26.6	142	28.4
Black	20	15.5	23	16.4	374	18.6	373	18.4	66	16.3	80	16.0
Mixed	2	1.6	2	1.4	44	2.2	44	2.2	7	1.7	9	1.8
Other	6	4.7	5	3.6	52	2.6	54	2.7	17	4.2	18	3.6
Missing	5	3.9	6	4.3	57	2.8	56	2.8	6	1.5	5	1.0
Age group												
18-39	15	11.6	16	11.4	99	4.9	96	4.7	41	10.1	58	11.6
40-59	46	35.7	49	35.0	505	25.2	505	25.0	178	43.8	210	42.0
60-79	60	46.5	65	46.4	1038	51.7	1050	51.9	179	44.1	222	44.4
80+	8	6.2	10	7.1	366	18.2	373	18.4	8	2.0	10	2.0
Waitlisted (dialysis only)												
Not listed	90	69.8	100	71.4	1709	85.1	1725	85.2	.	.	.	.
Listed	39	30.2	40	28.6	299	14.9	299	14.8	.	.	.	.
Month of positive test												
March	44	34.1	45	32.1	694	34.6	694	34.3	117	28.8	136	27.2
April	61	47.3	66	47.1	1053	52.4	1060	52.4	212	52.2	234	46.8
May	13	10.1	17	12.1	161	8.0	173	8.6	41	10.1	60	12.0
June	8	6.2	9	6.4	77	3.8	72	3.6	17	4.2	26	5.2
July	1	0.8	1	0.7	17	0.9	20	1.0	13	3.2	17	3.4
August	2	1.6	2	1.4	6	0.3	5	0.3	6	1.5	27	5.4
